# Use of clinical chromosomal microarray in Chinese patients with autism spectrum disorder—implications of a copy number variation involving *DPP10*

**DOI:** 10.1186/s13229-017-0136-x

**Published:** 2017-06-26

**Authors:** Annisa Shui Lam Mak, Annie Ting Gee Chiu, Gordon Ka Chun Leung, Christopher Chun Yu Mak, Yoyo Wing Yiu Chu, Gary Tsz Kin Mok, Wing Fai Tang, Kelvin Yuen Kwong Chan, Mary Hoi Yin Tang, Elizabeth Tak-Kwong Lau Yim, Kin Wai So, Victoria Qinchen Tao, Cheuk Wing Fung, Virginia Chun Nei Wong, Mohammed Uddin, So Lun Lee, Christian R. Marshall, Stephen W. Scherer, Anita Sik Yau Kan, Brian Hon Yin Chung

**Affiliations:** 1 Department of Obstetrics and Gynaecology, Queen Elizabeth Hospital, Hong Kong, Special Administrative Region, China; 2 Department of Paediatrics & Adolescent Medicine, LKS Faculty of Medicine, The University of Hong Kong, Hong Kong, Special Administrative Region, China; 3 Department of Obstetrics and Gynaecology, LKS Faculty of Medicine, The University of Hong Kong, Hong Kong, Special Administrative Region, China; 4 Department of Obstetrics and Gynaecology, Tsan Yuk Hospital, Hong Kong, Special Administrative Region, China; 5 Duchess of Kent Children’s Hospital, Hong Kong, Special Administrative Region, China; 6 The Centre for Applied Genomics, The Hospital for Sick Children, Toronto, Canada; 7 McLaughlin Centre and Department of Molecular Genetics, University of Toronto, Toronto, Canada

**Keywords:** Copy number variations (CNVs), Autism spectrum disorder (ASD), Array comparative genomic hybridization (aCGH), Chinese, *DPP10*

## Abstract

**Background:**

Array comparative genomic hybridization (aCGH) is recommended as a first-tier genetic test for children with autism spectrum disorder (ASD). However, interpretation of results can often be challenging partly due to the fact that copy number variants (CNVs) in non-European ASD patients are not well studied. To address this literature gap, we report the CNV findings in a cohort of Chinese children with ASD.

**Methods:**

DNA samples were obtained from 258 Chinese ASD patients recruited from a child assessment center between January 2011 and August 2014. aCGH was performed using NimbleGen-CGX-135k or Agilent-CGX 60k oligonucleotide array. Results were classified based on existing guidelines and literature.

**Results:**

Ten pathogenic CNVs and one likely pathogenic CNV were found in nine patients, with an overall diagnostic yield of 3.5%. A 138 kb duplication involving 3′ exons of *DPP10* (arr[GRCh37] 2q14.1(116534689_116672358)x3), reported to be associated with ASD, was identified in one patient (0.39%). The same CNV was reported as variant of uncertain significance (VUS) in DECIPHER database. Multiple individuals of typical development carrying a similar duplication were identified among our ancestry-matched control with a frequency of 6/653 (0.92%) as well as from literature and genomic databases.

**Conclusions:**

The *DPP10* duplication is likely a benign CNV polymorphism enriched in Southern Chinese with a population frequency of ~1%. This highlights the importance of using ancestry-matched controls in interpretation of aCGH findings.

**Electronic supplementary material:**

The online version of this article (doi:10.1186/s13229-017-0136-x) contains supplementary material, which is available to authorized users.

## Background

Autism spectrum disorders (ASD) refer to conditions characterized by impairment in social interaction and social communication, as well as restricted or repetitive behavior, interests, and activities [1]. ASD is well known to have a genetic basis, in particular, studies from the recent decade have shown that rare and fully penetrant copy number variations (CNVs) explain approximately 5–10% of the disorder depending on the cohort examined and the platform used for analysis [2], a yield that is substantially higher than conventional karyotyping [3]. Therefore, the latest guidelines by the International Standards for Cytogenomic Arrays (ISCA) Consortium has recommended chromosomal microarray as the first line investigation for children with ASD and various developmental disorders [4].

The interpretation of CNV pathogenicity is challenging and complicated by various factors. It has been shown that the diagnostic yield of array comparative genomic hybridization (aCGH) in a fixed cohort of pediatric patients with intellectual disability (ID) have increased from 19 to 31% in 2 years. Reasons for the reclassification of CNVs include emergence of new literature on known genes, discovery of new candidate genes, refinement of information, and remapping of gene location [5]. On the contrary, because of the rare nature of conditions encountered in clinical genetics, ascertainment bias due to small sample sizes or ancestry-related differences in CNV carrier frequency may occur [6]. In a recent study, Manrai et al. pointed out that the possibility of ruling out the pathogenicity of a population-enriched benign variant depends on the representation of specific population in the overall sample and that five variants previously classified as pathogenic for hypertrophic cardiomyopathy were misclassified because they were much more prevalent in African Americans, which were under-represented in previous studies [7]. Ascertainment bias is particularly challenging outside North America and Europe since most studies on CNV are focused on patients of European ancestry. Previous studies in general Asian populations have also shown substantial differences in terms of the location and frequency of CNVs across the genome [8].

CNV studies of ASD in Chinese patients have mostly been limited to case reports or candidate gene single nucleotide polymorphisms (SNPs) based on findings in ASD patients of European ancestry [9]. Using research array platforms and bioinformatics pipelines, Gazzellone et al. [10] and Yin et al. [11] reported on 104 and 335 ASD probands from Harbin of northern China and Taiwan, respectively. We examined the characteristics of CNVs in a southern Chinese cohort with clinically diagnosed ASD to address this knowledge gap. A clinical aCGH platform was used for the analysis. This gives the advantages of yielding clinically relevant results and utilization of comparable controls from our diagnostic laboratory service.

## Methods

### Patient recruitment

Patients diagnosed with ASD from the Department of Paediatrics and Adolescent Medicine, the University of Hong Kong, in the period from January 2011 to August 2014 were enrolled into the study. Diagnosis of ASD was made by developmental pediatrician or clinical psychologist using the Diagnostic and Statistical Manual of Mental Disorders, fourth edition, text revision (DSM IV-TR) or fifth edition (DSM-V). In difficult cases, tools such as Childhood Autism Rating Scale (CARS), Checklist for Autism in Toddlers (CHAT-23 modified) [12], Autism Diagnostic Interview Revised (ADIR), and Autism Diagnostic Observation Schedule (ADOS) were used. For the genetic evaluation, we followed the suggestion by Miller et al [4]. We offered aCGH to patients with unexplained ASD as the first-tier testing after review by a clinical geneticist. Clinically recognizable syndromic conditions would be confirmed with appropriate genetic test targeted to that specific condition, and aCGH would only be offered if the targeted testing showed negative results. We recruited 288 patients with ASD, and DNA was obtained from these individuals. Subsequently, DNA was also obtained from their parents in the post-aCGH genetic counseling session. The recruitment process did not discriminate between children of various ethnicities but our analysis focused on only 258 patients of self-reported Chinese ethnicity. Some of the patients reported in this study were also included in a previous publication describing the impact of aCGH on clinical management [13]. However, the current manuscript focuses only on patients with ASD and includes 116 additional ASD patients.

### Genotyping and variant calling

Out of the 288 patients, aCGH of 255 (88.5%) were done using Nimblegen CGX-135k oligonucleotide array, and 33 (11.5%) were done using Agilent-CGX 60k oligonucleotide array. Array processing and analysis was performed in the diagnostic laboratory of Tsan Yuk Hospital (TYH), which offers both prenatal and postnatal aCGH testing for patients in Hong Kong. Details of the testing methodology, variant calling, and interpretation have been previously reported [13].

Detected CNVs were systematically evaluated for their clinical significance by comparing the CNVs to information in the Signature Genomics’ proprietary Genoglyphix Chromosome Aberration Database (Signature Genomics, Spokane, WA, USA), internal laboratory database at TYH, and public databases (Database of Genomic Variant (DGV), ISCA Database, Children Hospital of Philadelphia database (CHOP), and Database of Chromosomal Imbalance and Phenotype in Humans using Ensembl Resources (DECIPHER)). Categorization of CNVs was based on available phenotypes and comparison of phenotypes with genes in the region of copy gain or loss. This was done through searching Online Mendelian Inheritance in Man (OMIM), PubMed, RefSeq, and the University of California Santa Cruz (UCSC) genome browser. CNVs were classified to (a) pathogenic, (b) variant of uncertain clinical significance (VUS) of likely pathogenic, (c) VUS of likely benign, (d) VUS with no subclassification, or (e) benign according to the 2013 American College of Medical Genetics & Genomics (ACMG) practice guideline [14]. Only pathogenic and likely pathogenic CNVs were regarded as clinically significant. TYH possesses an internal control database of 653 individuals of Chinese ethnicity with typical development and insignificant medical history determined by a self-administered questionnaire. Majority of the controls are parental samples (*n* = 554) that were primarily collected for prenatal diagnostic interpretation.

### Validation of CNVs

CNVs involving the 3′ exons of *DPP10* in the current study were confirmed by digital PCR, using the QuantStudio^TM^ 3D Digital PCR System platform and ProFlex PCR System (Life Technologies, CA). Taqman copy-number primer-probe assay was designed using GeneAssist^TM^ Copy Number Assay workflow builder. The reaction mix was loaded onto a QuantStudio^TM^ 3D Digital PCR 20K Chip using an automatic chip loader according to the manufacturer’s protocol. The chips underwent thermocycling on the ProFlex PCR System with the default conditions suggested by the primer-probe assay. After PCR, chip images were analyzed using QuantStudio^TM^ 3D instrument.

Quantitative real-time PCR (qPCR) was performed to more accurately delineate the breakpoints of the *DPP10* CNVs in our DNA samples. Ten primer assays, five at the proximal site and five at the distal site, were designed followed by qPCR using QuantiNova SYBR Green PCR Kit (Qiagen, Germany) on a 7900 Fast Real-time PCR System (Applied Biosystems, MA). Cycle threshold (Ct) value was called by the software RQ manager v1.2, and the relative copy number of the target assay was determined using comparative Ct method.

## Results

Out of the 258 Chinese patients recruited, 215 were male and 43 were female, with a male to female ratio 5:1. Their age ranged from 1.5 to 25 years old. As mentioned above, our analysis focused on the 258 patients of self-reported Chinese ancestry. Of these, parental blood samples were obtained from both parents in 24 cases, from mothers only in three cases, and from a father only in one case. Ten patients were siblings from five families, and the rest of the patients were unrelated.

We did not identify many known CNVs previously implicated in ASD. For example, there were no *PTCHD1*-related CNVs, and none overlapping the loci of 22q11.2, 1q21, 5q15.2, 7q11, 15q13, or 17p11.2 [15, 16]. Nevertheless, patient 2 did harbor a distal 16p11.2 deletion. In addition, there were also CNVs found in the regions of 16p13.3 and 6q26 reported by Gazzellone et al. [10] and Yin et al. [11], respectively.

### Clinically significant *CNV*s

All identified CNVs were classified according to the ACMG Guidelines [14, 17]. Ten pathogenic CNVs and one likely pathogenic CNV were found in nine patients, resulting in a diagnostic yield of 3.5% (9/258) (Table 1). We detected a total of 52 VUS in 43 patients (16.7%). Of these, 2 CNVs overlap with VUS and 6 had partial overlap with those reported as pathogenic CNVs in ISCA database [18], while 21 overlap with those reported as VUS in DECIPHER [19]. A summary table of all VUS is shown (see Additional file 1).

**Table 1:** Array results of patients with ASD having pathogenic and likely pathogenic CNVs. Genomic coordinates were listed in human assembly GRCh37/hg19. Genes in bold represent the OMIM genes. Data set is available from the ArrayExpress repository, accession numbers E-MTAB-5672 and E-MTAB-5665

Patient no.	Lab no.	Sex/age	CNV	Size/CNV	Representative gene(s) within the CNV	Classification	Inheritance	Clinical features
1	A0572B	M/4 years	arr[GRCh37] 7q31.1(110978196_111337169)x1	0.36 Mb deletion	***IMMP2L***	VUS	Paternally inherited	High-functioning ASD
			arr[GRCh37] 15q25.2q26.1(84642246_91626219)x1	6.98 Mb deletion	***ADAMTSL3***, *UBE2Q2L*, *GOLGA6L4*, *ZSCAN2*, ***WDR73***, ***NMB***, *SEC11A*, ***ZNF592***, *ALPK3*, ***SLC28A1***, ***PDE8A***, *GOLGA6L3*, ***AKAP13***, *KLHL25*, ***AGBL1***, ***NTRK3***, ***MRPL46***, ***MRPS11***, ***DET1***, ***AEN***, ***ISG20***, ***ACAN***, *HAPLN3*, ***MFGE8***, ***ABHD2***, ***RLBP1***, ***FANCI***, ***POLG***, ***RHCG***, ***TICRR***, ***KIF7***, ***PLIN1***, ***PEX11A***, *WDR93*, ***MESP1***, ***MESP2***, ***ANPEP***, *C15orf38*, ***AP3S2***, ***ARPIN***, *ZNF710*, ***IDH2***, *SEMA4B*, ***CIB1***, *GDPGP1*, ***NGRN***, *ZNF774*, ***IQGAP1***, ***CRTC3***, ***BLM***, ***FURIN***, ***FES***, ***MAN2A2***, ***UNC45A***, *HDDC3*, *RCCD1*, ***PRC1***, ***VPS33B***	Pathogenic	De novo	
2	AGG0013	M/18 months	arr[GRCh37] 16p11.2(28488491_29046284)x1	0.56 Mb deletion	***CLN3***, ***APOBR***, ***IL27***, ***NUPR1***, ***SGF29***, ***SULT1A2***, ***SULT1A1***, *NPIPB8*, ***EIF3C***, *EIF3CL*, *NPIP89*, ***ATXN2L***, ***TUFM***, ***SH2B1***, ***ATP2A1***, ***RABEP2***, ***CD19***, ***NFATC21P***, ***SPNS1***, ***LAT***	Pathogenic	De novo	GDD, ASD
3	AGG0207	F/32 months	arr[GRCh37] Xp22.33(296519_4021220)x1	3.72 Mb deletion	***PPP2R3B***, ***SHOX***, ***CRLF2***, ***CSF2RA***, ***IL3RA***, ***SLC25A6***, ***ASMTL***, ***P2RY8***, ***AKAP17A***, ***ASMT***, *DHRSX*, ***ZBED1***, ***CD99***, ***XG***, ***GYG2***, ***ARSD***, ***ARSE***, ***ARSH***, ***ARSF***, ***MXRA5***, ***PRKX***	Pathogenic	Not maternally inherited	Short stature, GDD, ASD
4	AGG0238	M/29 months	arr(X)x2,(Y)x1	XXY	(Klinefelter syndrome)	Pathogenic	De novo	Developmental language delay, ASD with ADHD features
5	AGG0318	F/26 months	arr[GRCh37] 1q44(246807340_24920810﻿5)x1	2.40 Mb deletion	***CNST***, *SCCPDH*, ***AHCTF1***, ***ZNF695***, *ZNF670*, *ZNF669*, *C1orf229*, ***ZNF124***, *VN1R5*, ***ZNF496***, ***NLRP3***, *GCSAML*, *TRIM58*, *LYPD8*, *SH3BP5L*, *ZNF672*, *ZNF692*, *PGBD2*	Pathogenic	Inheritance not known; unbalanced translocation t(1;8)	ASD
			arr[GRCh37] 8q24.22q24.3(135567463_146304022)x3	10.76 Mb duplication	***ZFAT***, ***KHDRBS3***, *FAM135B*, ***COL22A1***, ***KCNK9***, ***TRAPPC9***, ***CHRAC1***, ***AGO2***, ***PTK2***, *DENND3*, *SLC45A4*, ***GPR20***, ***PTP4A3***, *MROH5*, *TSNARE1*, ***ADGRB1***, ***ARC***, ***JRK***, ***PSCA***, ***LY6K***, *THEM6*, ***SLURP1***, *LYPD2*, ***LYNX1***, ***LY6D***, ***GML***, ***CYP11B1***, ***CYP11B2***, ***LY6E***, *C8orf31*, ***LY6H***, ***GPIHBP1***, *ZFP41*, ***GLI4***, *ZNF696*, ***TOP1MT***, ***RHPN1***, ***MAFA***, *ZC3H3*, *GSDMD*, *MROH6*, ***NAPRT***, ***EEF1D***, *TIGD5*, ***PYCRL***, ***TSTA3***, *ZNF623*, *ZNF707*, *CCDC166*, *MAPK15*, ***FAM83H***, ***SCRIB***, ***PUF60***, ***NRBP2***, ***EPPK1***, ***PLEC***, ***PARP10***, ***GRINA***, ***SPATC1***, ***OPLAH***, ***EXOSC4***, ***GPAA1***, ***CYC1***, ***SHARPIN***, ***MAF1***, *WDR97*, *HGH1*, ***BOP1***, ***SCX***, ***HSF1***, ***DGAT1***, ***SCRT1***, *TMEM249*, ***FBXL6***, ***SLC52A2***, *ADCK5*, ***CPSF1***, ***SLC39A4***, ***VPS28***, ***TONSL***, ***CYHR1***, *KIFC2*, ***FOXH1***, ***PPP1R16A***, ***GPT***, *MFSD3*, ***RECQL4***, *LRRC14*, *LRRC24*, *C8orf82*, ***ARHGAP39***, *ZNF251*, ***ZNF34***, ***RPL8***, *ZNF517*, ***ZNF7***, ***COMMD5***, *ZNF250*, ***ZNF16***, *C8orf33*	Pathogenic		
6	AGG0126	M/29 months	arr[GRCh37] 6q26(162621987_163095834)x3	0.47 Mb duplication	***PARK2***	likely pathogenic	De novo	Speech delay; ASD; Hypospadias
7	AGG0128	M/23 months	arr[GRCh37] 16p13.11(15125758_16287903)x3	1.16 Mb duplication	***PDXDC1***, ***NTAN1***, ***RRN3***, *MPV17L*, *C16orf45*, ***KIAA0430***, ***NDE1***, ***MYH11***, *FOPNL*, ***ABCC1***, ***ABCC6***	Pathogenic	Maternally inherited	ASD
8	AGG0228	M/17 years	arr[GRCh37] 16p13.11(15125758_16287903)x3	1.16 Mb duplication	***PDXDC1***, ***NTAN1***, ***RRN3***, *MPV17L*, *C16orf45*, ***KIAA0430***, ***NDE1***, ***MYH11***, *FOPNL*, ***ABCC1***, ***ABCC6***	Pathogenic	Inheritance not known	Epilepsy; GDD; ASD
9	AGG0437	M/29 months	arr[GRCh37] 11p11.2(48103669_48388756)x2~3	0.29 Mb duplication (mosaic)	***PTPRJ***, *OR4B1*, *OR4X2*, *OR4ZX1*, *OR4S1*, *OR4C3*, *OR4C45*	Pathogenic	Inheritance not known	ASD
			arr[GRCh37] 11q12.1q12.2(55896790_61443324)x2~3	5.55 Mb duplication (mosaic)	***LRRC55***, ***APLNR***, ***TNKS1BP1***, ***SSRP1***, ***P2RX3***, ***PRG3***, ***PRG2***, *SLC43A3*, ***RTN4RL2***, ***SLC43A1***, ***TIMM10***, ***SMTNL1***, ***UBE2L6***, ***SERPING1***, *TPEL4*, ***CLP1***, ***ZDHHC5***, ***MED19***, ***TMX2*** *.* ***C11orf31***, *BTBD18*, ***CTNND1***, ***LPXN***, *ZFP91*, ***CNTF***, ***GLYAT***, ***GLYATL2***, *AK294973*, ***GLYALT1***, ***FAM111B***, ***FAM111A***, ***DTX4***, ***MPEG1***, ***OSBP***, ***PATL1***, ***STX3***, ***MRPL16***, ***GIF***, ***TCN1***, *AB231702*, *PLAC1L*, ***CCDC86***, ***PTGDR2***, ***ZP1*** *,* ***PRPF19***, *TMEM109*, *TMEM132A*, ***SLC15A3***, ***CD6***, ***VPS37C***, ***PGA3***, ***PGA4***, ***PGA5***, ***VWCE***, ***DDB1***, ***TKFC***, *CYB561A3*, ***TMEM138***, ***TMEM216***, *CPSF7*, ***SDHAF2***, *PPP1R32*, *LRRC10B*, ***ZYT7***	Pathogenic	Inheritance not known	

Patient 1 presented with high-functioning autism at the age of 4 years. There was no associated dysmorphism or physical anomaly. His parents were non-consanguineous, and there was a strong family history of high-achieving paternal male relatives with weak social skills. He was found to have two CNVs—a paternally inherited 0.36 Mb deletion at 7q31.1 classified as a VUS, as well as a de novo pathogenic 6.98 Mb deletion encompassing 15q25.2-26.1. The smaller 7q31.1 deletion encompasses *IMMP2L*, a gene whose SNPs were shown to be associated with ASD in a predominantly Caucasian study [20] but not in another study with northeastern Han Chinese [21]. On the other hand, the larger 15q25.2-26.1 deletion involved up to 40 OMIM genes, and the deletion is in close proximity with the more distal 15q26 region which is associated with neurobehavioral phenotype including ASD, attention deficit hyperactivity disorder (ADHD), learning difficulty, and hearing impairment [22].

Patient 2 presented with global developmental delay (GDD) and ASD at 18 months of age, and subsequently demonstrated satisfactory catch up in developmental milestones and autistic features. He had mildly elongated and expressionless face with open mouth appearance and downturned angles of mouth. His parents were non-consanguineous. Apart from the presence of specific language impairment in his elder brother, there was no family history of neuropsychiatric illness. He was found to have a de novo 0.56 Mb distal 16p11.2 microdeletion which not only explains his neurodevelopmental phenotype, but is also associated with obesity [23]. Identification of the deletion facilitated the need to conduct regular weight surveillance, highlighting the importance of genetic testing in anticipatory management of genomic conditions. Despite the positive history of specific language impairment, his brother was tested negative for the 16p11.2 deletion, underscoring the genetic heterogeneity of ASD and that in ~70% of families with multiple affected siblings, the affected children might carry different ASD-relevant mutations or CNVs [24].

Patient 3 presented at 32 months of age with GDD and ASD, in association with soft dysmorphic features including telecanthus, small low-set ears with slightly thickened helices, and slightly shortened forearms. Her height was normal at presentation but fell from the 25th to 3rd percentile within the subsequent year. Further questioning revealed that there was also antenatal history of short long bones, which was commented to be normal post-delivery. Her parents were non-consanguineous and there was no family history of neurodevelopmental disorders. She is now studying in a school for children with mild ID. Her microarray showed a pathogenic Xp22.33 deletion measuring 3.72 Mb that encompassed both *ASMT* and *SHOX*, which can explain the clinical features of the patient. The mother was tested negative for the CNV while paternal information was not available. The *ASMT* gene was shown to be associated with social impairment and autistic traits [25], whereas the *SHOX* gene is known to be associated with short stature. Together, the two genes explained her overall clinical picture. The findings prompted further radiographic assessment, which showed mild Madelung deformity of her forearms, reaffirming the microarray findings. Despite being eligible for receiving growth hormone therapy under public healthcare funding, her parents opted not for treatment as they were satisfied with the overall growth of the child. Again, this patient revealed the importance of understanding the underlying molecular defects prompting targeted surveillance of possible complications. Her developmental progress was reassessed at 5 years of age, showing persistence of ASD with mild ID. She is scheduled to enter school for children with mild ID.

Patient 4 presented with GDD at 18 months and was diagnosed to have ASD in early childhood. On subsequent visits he was also noted to have hyperactivity features. His intellectual ability caught up gradually with above-average academic performance in mainstream school. His parents were non-consanguineous, and apart from his twin brother who was diagnosed with developmental language delay, there was no family history of neurodevelopmental disorder. For both the patient and his twin brother, aCGH showed XXY, which was indicative of Klinefelter syndrome. The association between Klinefelter syndrome and ASD has been previously described [26]. He was also followed up by endocrinologists for potential complications of the syndrome.

Patient 5 presented with language delay with mild autistic features at 18 months. Further assessment at 26 months confirmed the diagnosis of ASD. At 6 years 3 months, assessment revealed borderline ID with persistence of ASD features. Her parents were non-consanguineous, and there was no family history of neurodevelopmental disorder. She was found to have 1q44 deletion of 2.40 Mb as well as 8q24.22q24.3 duplication of 10.76 Mb, which was associated with GDD in the DECIPHER database and implicated in the autism endophenotype of social responsiveness in genome-wide quantitative linkage analyses respectively [27]. Such results prompted further testing with karyotype and fluorescence in situ hybridization (FISH), which confirmed that the changes were due to an unbalanced translocation. Parental information was not available.

Patient 6 presented with language delay, ASD at 22 months. There was no dysmorphic feature except the presence of penile hypospadias requiring surgical repair, and his cognitive function was normal. His parents were non-consanguineous and there was no family history of neurodevelopmental disorder. He was found to have a de novo 0.47 Mb duplication of likely pathogenic as it involved the *PARK2* gene. The gene encodes for an ubiquitin protein ligase, which was first discovered in early onset Parkinson disease with autosomal recessive mutations [28] and later found to be associated with CNVs in schizophrenia [29] and neurodevelopmental disorders including ADHD [30] and ASD [15]. CNV involving this region was the focus of a previous publication from Yin et al, where it was found in 4 out of 335 ASD patients of Han Chinese descent [11].

Both patients 7 and 8 were diagnosed with ASD but with different neurodevelopmental outcome. Patient 7 had a history of suspected neonatal seizure in association with a small subdural hematoma possibly secondary to traumatic delivery. Subsequent magnetic resonance imaging (MRI) of the brain was normal. He presented with language delay and autistic features at 17 months and was diagnosed with ASD at 23 months. At the age of 5, cognitive assessment revealed an average intelligence quotient with signs of dyslexia. No major challenging behavior was detected. On the other hand, patient 8 presented during preschool years with GDD and autistic features and was subsequently diagnosed to have ASD as well as moderate ID. He also had adolescent-onset epilepsy requiring long-term use of sodium valproate. MRI of the brain showed a static arachnoid cyst at the left anterior middle cranial fossa which was managed conservatively. However, he developed significant behavioral difficulties at adolescence requiring intensive management by the psychiatrists. Both patients came from families with non-consanguineous parents and without family history of neurodevelopmental disorders. Their aCGH results both showed 1.16 Mb duplications at 16p13.11. Despite previous concerns regarding the possibility of this CNV being a benign variant [31], further studies have provided evidence of its pathogenicity in multiple neuropsychiatric conditions including ASD, ADHD, ID, and schizophrenia [32–34]. Epilepsy has also been reported in association with 16p13.11 duplication [34].

Patient 9 presented with impaired social interaction at 29 months and was diagnosed with ASD. There was no associated physical anomaly, and his cognitive function was normal. His parents were non-consanguineous, and there was no family history of neurodevelopmental disorders. He was found to have two consecutive duplication in mosaic pattern, 11q12.1q12.2 (5.55 Mb) and 11p11.2 (0.29 Mb), spanning across the centromere. The result was suggestive of a marker chromosome, which was later confirmed by the karyotype mos 47,XY,+mar[8]/46,XY[52]. No case was previously reported in the literature but there was one entry (Variant ID: NSSV582454) in the ISCA database [35] with similar duplication size of 3.67 Mb, presenting as GDD and delayed speech and language development.

### *DPP10* duplication—a CNV polymorphism enriched in Chinese population

**Fig. 1:**
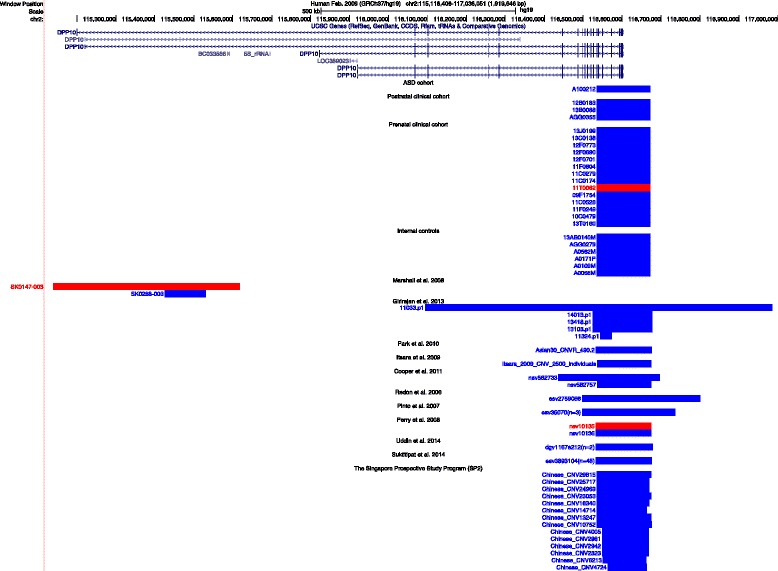
Genomic browser figure showing CNVs overlapping *DPP10* in various databases. This figure was modified from the USCS genome browser (https://genome.ucsc.edu). Different isoforms of CNVs containing *DPP10* reported in literature, as well as CNVs from relevant databases of at least 50% physical overlap to the one identified in our ASD cohort are included. *Red* and *blue bars* represent deletions and duplications, respectively. Subjects from the TYH database are represented at the top of the figure; ASD subjects reported by Girirajan et al. and Marshall et al. are represented in the middle part of the figure. Control subjects reported in Database of Genomic Variants (DGV) study are represented at the lower part of the figure. Case numbers are consistent throughout the article, table, and supplemented documents

A Chinese-enriched CNV polymorphism was identified at the 3′ exons of *DPP10*, a gene previously speculated to have a role in ASD [36, 37]. *DPP10* encodes for dipeptidyl peptidase like 10. *DPP10* has at least five transcript variants, and the primary transcript has 26 exons. The gene was previously implicated in ASD susceptibility in at least two large independent studies (See Fig. 1). Within our cohort, we identified only one 0.14 Mb duplication arr[GRCh37] 2q14.1(11653﻿4689_116672358)x3 overlapping exons 14 to 26 of *DPP10* (NM_020868.3) in a 17-year-old patient (patient 10). Other than ASD, he also had catatonia and mild ID. Parental aCGH testing was declined (see Fig. 1).

This CNV involving *DPP10* has been reported as a VUS (ID: 287659) in DECIPHER with 50% reciprocal overlap. Two studies have reported CNVs associated with *DPP10* before. Girirajan et al. [37] identified five patients with duplications among 2588 cases, three of whom had more than 50% reciprocal overlap with patient 10, and the remaining two only partially overlap. On the other hand, Marshall et al. [36] identified two CNVs (one deletion and one duplication) in 427 unrelated ASD probands, both of which did not overlap with the CNV identified in our cohort. The CNVs associated with *DPP10* reported in the above two studies are listed in Additional file 2. No such duplication was found in any of the controls from the above projects, which comprised of 500 individuals from the German PopGen project, 1152 from Canada, 207 from the National Institute of Mental Health (NIMH) control cohort with exclusively European origins, and 373 individuals from ClinSeq comprising of more than 90% Caucasians [38].

Despite these findings, within the local laboratory database, we found similar duplications in 17 other patients (3 postnatal cases and 14 prenatal cases). The clinical indications were heterogeneous, and recruitment and follow up of these patients were not done systematically and therefore would be subjected to different ascertainment bias. Nevertheless, as far as we can ascertain, none of these patients had evidence of ASD.

To describe the details of these patients further, among the three patients found in the postnatal cohort (*n* = 3/441); there was one patient with mild developmental delay, one with short stature but normal development, and one with dysmorphic features, hypotonia during neonatal period, and subsequently normal development. Non-ASD individuals with the CNV overlapping *DPP10* are listed (see Additional file 3). All of the above three patients had *DPP10* duplication. In the prenatal cohort (*n* = 14/1150) on the other hand, referral indications were as follows: (i) stillbirth (*n* = 1), (ii) abnormal antenatal ultrasound (*n* = 6), (iii) abnormal aneuploidy screening (*n* = 5), of which three were later confirmed with trisomy 21, and (iv) previous children with aneuploidy (*n* = 2). In total, 13 *DPP10* duplications and one deletion (*n* = 14) of the same size and genomic location were identified in the prenatal group. The clinical outcomes of these 14 pregnancies included one stillbirth and three terminations of pregnancies, which included two of the three cases with both trisomy 21 and *DPP10* duplication. The other ten were liveborns (71.4%), and their development was followed up routinely in the Maternal and Child Health Centre. Besides the remaining child with trisomy 21 in addition to the *DPP10* duplication, one additional child was found to have GDD and one other has mild attention problem (7.1%).

In order to estimate the prevalence of the *DPP10* duplication in Hong Kong, we examined the internal control database of TYH, which consisted of 653 Chinese of typical development, and their DNA samples were genotyped and analyzed using the same methods as our positive cases. Six out of 653 individuals were identified to have the similar *DPP10* duplications (0.92%). When comparing the prevalence of *DPP10* duplications in ASD cases (1/258) and controls (6/653), the *p* value did not infer statistical significance (*p* = 0.68, two-tailed Fisher’s test) to suggest any causative nature of *DPP10* duplication in ASD.

Molecularly, we validated and mapped the breakpoints in 24 different samples carrying the *DPP10* duplication (and deletion) by qPCR and found that both the 5′ and 3′ ends consistently map to the same regions. By the same method of qPCR, the breakpoints of the CNV were narrowed down further from hg[19]chr2:116524182-116529245 proximally to hg[19]chr2:116672358-116677229 distally.

The ancestry-related difference in the background prevalence of *DPP10* duplication was further studied by literature and database search. Park et al. [8] sought to identify common Asian CNVs. Interestingly, in their study, one *DPP10* duplication of size and location comparable to our patient was found in one out of ten Han Chinese subjects but not in subjects of Japanese or Korean descent. Extensive search of regional databases, various studies [39–44] extracted from DGV [45], the cohort of Itsara et al. [46], and the Singapore Prospective Study Program (SP2) [47, 48] was performed. Seventy-two individuals with typical development and CNVs containing *DPP10* of at least 50% physical overlap the one identified in our ASD cohort were identified. Individuals with the CNV are listed (see Additional file 4). Within the SP2 cohort, where ethnicity is available, the duplication was found in 14/2857 (0.49%) individuals of Chinese origin.

## Discussion

This study adds to the growing number of CNV studies in ASD in Chinese and is to our knowledge the third study with over a hundred patients. The first two studies were by Gazzellone et al. [10] on 104 Chinese from Harbin and Yin et al. who reported the CNV findings in a clinical ASD cohort of 335 Han Chinese (with a replication cohort of 301 ASD individuals) from Taiwan [11].

Gazzellone et al. identified at least one de novo rare CNV in 8.6% of his cohort whereas Yin et al. found several well-known ASD-associated CNVs from other ASD populations (e.g., 1q21.1, 15q11.21-13.1, 15q13.3, 16p11.2, 22q11.2, 22q13.33) in 5.1% Taiwan Chinese with ASD. The diagnostic yield of our cohort in Hong Kong is 3.5% but direct comparison is difficult as our study is the only one among the three that uses a clinical aCGH platform and reports only pathogenic/likely pathogenic CNVs using stringent clinical laboratory criteria [13]. We did not identify many known ASD-associated CNVs, perhaps due to the limited sample size of the cohort.

Although there were some overlap of our findings with these studies, such as 16p11.2 deletion and CNV involving *PARK2*, the results of these three studies substantiate the extensive genetic heterogeneity that is inherent to ASD even within the Chinese population. Further work in this ethnic group may identify new population-enriched variants that contribute to ASD, e.g., *PARK2* locus from Yin et al. On the other hand, identification of ethnic specific loci, e.g., *DPP10* duplication from our study and the 24 kb partial *YWHAE* duplication reported by Gazzellone et al. [10], will also avoid over-calling of pathogenic CNVs.

In addition to the duplication in patient 6, we identified two smaller deletions involving *PARK2* in two patients not known to be affected with ASD in our database. One was found in a patient with GDD, hypotonia and a family history of ID (lab number AGG0151, arr[GRCh37] 6q26(162740393_162865594)x1). The other was found in a patient with dysmorphism, short stature, and GDD (lab number AGG0359, arr[GRCh37] 6q26(162687401_162901598)x1) and was concurrently found to have Russell Silver syndrome (RSS) due to maternal uniparental disomy 7. While the duplication of patient 6 involved exons 2–4, the other two deletions overlap exon 2 only. Both deletions and duplications involving *PARK2* have been associated with ASD with variable expressivity and incomplete penetrance [11, 49]. When considering the pathogenicity, the size of the CNV, exons involved, and whether it was a copy gain or loss should be considered. It has been suggested that CNVs affecting exons 5–12 result in a more severe phenotype than those affecting exons 2–4. On the other hand, within exons 2–4, duplications lead to a more severe phenotype than deletions, due to greater interference to gene expression [11, 49]. Our findings in this cohort echo the above observations in that the largest duplication in *PARK2* was indeed found in a patient with ASD, while the smaller deletions involving only exon 2 were identified in non-ASD patients. As we only have one ASD patient with a CNV involving *PARK2*, the number is too small to make any conclusion about the disease causing nature of this CNV. However, this is in line with the suggestion that *PARK2* is a candidate gene and should at least be considered as a susceptibility locus.

The most important finding in this study is the identification of a ~135 kb *DPP10* duplication enclosing the 3′ exons of *DPP10* as a polymorphism frequently seen in Chinese population. Given the previous literature on large case-control studies, we initially concluded that the *DPP10* duplication could be significant and represent an ASD susceptibility locus. We explored this further by performing a search in our internal CNV database for *DPP10* CNVs. To our surprise, we were able to identify 24 such CNVs (23 duplications and 1 deletion) in our database of nearly 2000 samples (controls in combination), in contrast to not a single *DPP10* CNV found in nearly 2000 control subjects from Marshall’s and Girirajan’s studies combined. We also noted that for most of the *DPP10* duplications, their 3′ and 5′ ends consistently map to the same regions (Fig. 1). The location of the breakpoints of these CNV in our database was confirmed by qPCR. We could not identify any segmental duplication or other signature associated with homology-based sequence recombination around these breakpoints. These findings suggest that these *DPP10* duplications likely originate from the same ancestral event in the Chinese population.

A careful comparison of the prevalence of *DPP10* duplications in our ASD case (1/258) with control (6/653) showed no statistically significant difference (*p* = 0.68), suggesting this duplication is not likely to be pathogenic. The benign nature of this duplication is especially likely as it is a partial duplication with a breakpoint lying outside the last exon of the gene. Such postulation is further supported by findings from our database search including SP2, which archive normal populations of Chinese ethnicity.

Caution, however, should be exercised when making broader interpretations regarding the pathogenicity of the *DPP10* in ASD. While the 138 kb duplication in our study, mapped to the 3′ end of *DPP10*, might be a benign polymorphism, our findings do not exclude the possibility that other genomic variants involving *DPP10* can still have functional impact on neuronal potassium channel gating, and hence contribute to the pathogenesis of ASD and other neurodevelopmental disorders. Furthermore, there is a possibility that the *DPP10* duplication in our study may still confer risk for ASD and related disorders and should be considered in context. As an example, chromosome 15q11.2 (breakpoint_1 to breakpoint_2) deletion is a well-known CNV that can be found in both patients with autism or schizophrenia and normal population. Recent careful neurocognitive profiling has shown that 15q11.2 deletion can confer cognitive deficits with accompanying MRI changes even in control subjects [50], illustrating the complexity in interpreting the contribution of CNVs in neurocognitive phenotypes.

Nonetheless, this finding of CNVs overlapping *DPP10* illustrates the importance of using ancestry-matched controls and keeping a local internal control database when characterizing the clinical relevance of rare variants in a population. This is important in the evaluation of children with ASD and, perhaps, even more important in prenatal diagnosis as diagnostic uncertainties may impact on the reproductive decisions of couples, resulting in unnecessary termination of pregnancy.

### Advantages, limitations, and future directions

There are two main advantages to this study. This is the first study on the clinical application of aCGH in ASD individuals in a predominantly Southern Chinese population; this is also the first and largest single-center study reporting *DPP10* CNV in normal control subjects, supported by extensive literature/database search.

Our study has several limitations. The size of our cohort was small for us to perform comparison of CNV burden (call rate and length) between patients and controls. Furthermore, only a small percentage of family members consented for parental aCGH testing and hence, there was insufficient information on the inheritance, i.e., inherited or de novo. Our clinical setting and lack of research capacities limited us to perform further functional studies of our findings, such as the brain expression pattern of *DPP10*. Although seven children born with a prenatal diagnosis of *DPP10* duplication were found to have typical development from routine child health/development screening, it will be interesting to prospectively follow-up the full cohort with comprehensive neurodevelopmental assessment to assess the phenotypic expressivity and penetrance of this CNV commonly found in Chinese. It would also be of research value to narrow down the precise breakpoints of the duplication and clone it to a simple robust assay for population screening.

## Conclusion

We found ten pathogenic and one likely pathogenic CNV in nine patients of Chinese ancestry, resulting in a diagnostic yield of 3.5% (9/258). Majority of these CNVs did not overlap with those previously reported to be implicated in ASD (22q11.2, 1q21, 5q15.2 etc.). We also illustrated that the DPP10 duplication mapped to the 3′ end is likely a benign CNV polymorphism enriched in Southern Chinese with a population frequency of ~1%, despite previous reports of its role in ASD. Hence caution should be taken when making interpretations of aCGH findings, and ancestry-matched controls should be used to accurately delineate CNV pathogenicity.

## Additional files

Additional file 1: Table S1. Regions of variants of uncertain clinical significance found in our cohort. Genomic coordinates were listed in the human assembly GRCh37/hg19. (DOCX 18 kb)

Additional file 2: Table S2. Coordinates [hg19] of CNVs overlapping *DPP10* in chromosome 2 in Marshall et al., Girirajan et al., and the DECIPHER database. (DOCX 19 kb)

Additional file 3: Table S3. Indications of testing in non-ASD individuals with CNVs overlapping *DPP10* in TYH database. (DOCX 15 kb)

Additional file 4: Table S4. Coordinates in [hg19] of similar CNVs overlapping *DPP10* in the Database of Genomic Variants (DGV) study, cohort in the study by Itsara et al., Singapore Genome Variation Project (SGVP) (43), Singapore Prospective Study Program (SP2), and a Chinese individual described by Park et al. (DOCX 19 kb)

## Abbreviations

aCGH: Array comparative genomic hybridization
ADIR: Autism diagnostic interview-revised
ADHD: Attention deficit hyperactivity disorder
ASD: Autism spectrum disorder
ADOS: Autism diagnostic observation schedule
CARS: Childhood autism rating scale
CNV: Copy number variation
Ct: threshold cycle
DSM: Diagnostic and statistical manual of mental disorders
GDD: Global developmental delay
ID: Intellectual disability
qPCR: quantitative polymerase chain reaction
SNPs: Single nucleotide polymorphisms
TYH: Tsan Yuk Hospital
VUS: Variant of uncertain clinical significance

## References

[1] American Psychiatric Association (2013). Diagnostic and statistical manual of mental disorders (DSM-5®).

[2] Schaefer GB, Mendelsohn NJ, Professional P, Guidelines C (2013). Clinical genetics evaluation in identifying the etiology of autism spectrum disorders: 2013 guideline revisions. Genet Med.

[3] Shen Y, Dies KA, Holm IA, Bridgemohan C, Sobeih MM, Caronna EB, Miller KJ, Frazier JA, Silverstein I, Picker J (2010). Clinical genetic testing for patients with autism spectrum disorders. Pediatrics.

[4] Miller DT, Adam MP, Aradhya S, Biesecker LG, Brothman AR, Carter NP, Church DM, Crolla JA, Eichler EE, Epstein CJ (2010). Consensus statement: chromosomal microarray is a first-tier clinical diagnostic test for individuals with developmental disabilities or congenital anomalies. Am J Hum Genet.

[5] Palmer E, Speirs H, Taylor PJ, Mullan G, Turner G, Einfeld S, Tonge B, Mowat D (2014). Changing interpretation of chromosomal microarray over time in a community cohort with intellectual disability. Am J Med Genet A.

[6] Kamien B, Lionel AC, Bain N, Scherer SW, Hunter M (2014). Outfoxed by RBFOX1-a caution about ascertainment bias. Am J Med Genet A.

[7] Manrai AK, Funke BH, Rehm HL, Olesen MS, Maron BA, Szolovits P, Margulies DM, Loscalzo J, Kohane IS (2016). Genetic misdiagnoses and the potential for health disparities. N Engl J Med.

[8] Park H, Kim JI, Ju YS, Gokcumen O, Mills RE, Kim S, Lee S, Suh D, Hong D, Kang HP (2010). Discovery of common Asian copy number variants using integrated high-resolution array CGH and massively parallel DNA sequencing. Nat Genet.

[9] Chung BH-Y, Tao VQ, Tso WW-Y (2014). Copy number variation and autism: new insights and clinical implications. J Formos Med Assoc.

[10] Gazzellone MJ, Zhou X, Lionel AC, Uddin M, Thiruvahindrapuram B, Liang S, Sun C, Wang J, Zou M, Tammimies K (2014). Copy number variation in Han Chinese individuals with autism spectrum disorder. J Neurodev Disord.

[11] Yin CL, Chen HI, Li LH, Chien YL, Liao HM, Chou MC, Chou WJ, Tsai WC, Chiu YN, Wu YY (2016). Genome-wide analysis of copy number variations identifies PARK2 as a candidate gene for autism spectrum disorder. Mol Autism.

[12] Wong V, Hui LH, Lee WC, Leung LS, Ho PK, Lau WL, Fung CW, Chung B (2004). A modified screening tool for autism (Checklist for Autism in Toddlers [CHAT-23]) for Chinese children. Pediatrics.

[13] Tao VQ, Chan KY, Chu YW, Mok GT, Tan TY, Yang W, Lee SL, Tang WF, Tso WW, Lau ET (2014). The clinical impact of chromosomal microarray on paediatric care in Hong Kong. PLoS One.

[14] South ST, Lee C, Lamb AN, Higgins AW, Kearney HM, Working Group for the American College of Medical G, Genomics Laboratory Quality Assurance C (2013). ACMG Standards and Guidelines for constitutional cytogenomic microarray analysis, including postnatal and prenatal applications: revision 2013. Genet Med.

[15] Glessner JT, Wang K, Cai G, Korvatska O, Kim CE, Wood S, Zhang H, Estes A, Brune CW, Bradfield JP (2009). Autism genome-wide copy number variation reveals ubiquitin and neuronal genes. Nature.

[16] Sorte HS, Gjevik E, Sponheim E, Eiklid KL, Rodningen OK (2013). Copy number variation findings among 50 children and adolescents with autism spectrum disorder. Psychiatr Genet.

[17] Kearney HM, Thorland EC, Brown KK, Quintero-Rivera F, South ST (2011). American College of Medical Genetics standards and guidelines for interpretation and reporting of postnatal constitutional copy number variants. Genet Med.

[18] Kaminsky EB, Kaul V, Paschall J, Church DM, Bunke B, Kunig D, Moreno-De-Luca D, Moreno-De-Luca A, Mulle JG, Warren ST (2011). An evidence-based approach to establish the functional and clinical significance of copy number variants in intellectual and developmental disabilities. Genet Med.

[19] Bragin E, Chatzimichali EA, Wright CF, Hurles ME, Firth HV, Bevan AP, Swaminathan GJ (2014). DECIPHER: database for the interpretation of phenotype-linked plausibly pathogenic sequence and copy-number variation. Nucleic Acids Res.

[20] Maestrini E, Pagnamenta AT, Lamb JA, Bacchelli E, Sykes NH, Sousa I, Toma C, Barnby G, Butler H, Winchester L (2010). High-density SNP association study and copy number variation analysis of the AUTS1 and AUTS5 loci implicate the IMMP2L-DOCK4 gene region in autism susceptibility. Mol Psychiatry.

[21] Liang S, Wang XL, Zou MY, Wang H, Zhou X, Sun CH, Xia W, Wu LJ, Fujisawa TX, Tomoda A (2014). Family-based association study of ZNF533, DOCK4 and IMMP2L gene polymorphisms linked to autism in a northeastern Chinese Han population. J Zhejiang Univ Sci B.

[22] Poot M, Verrijn Stuart AA, van Daalen E, van Iperen A, van Binsbergen E, Hochstenbach R (2013). Variable behavioural phenotypes of patients with monosomies of 15q26 and a review of 16 cases. Eur J Med Genet.

[23] Bachmann-Gagescu R, Mefford HC, Cowan C, Glew GM, Hing AV, Wallace S, Bader PI, Hamati A, Reitnauer PJ, Smith R (2010). Recurrent 200-kb deletions of 16p11.2 that include the SH2B1 gene are associated with developmental delay and obesity. Genet Med.

[24] Yuen RK, Thiruvahindrapuram B, Merico D, Walker S, Tammimies K, Hoang N, Chrysler C, Nalpathamkalam T, Pellecchia G, Liu Y (2015). Whole-genome sequencing of quartet families with autism spectrum disorder. Nat Med.

[25] Jonsson L, Anckarsater H, Zettergren A, Westberg L, Walum H, Lundstrom S, Larsson H, Lichtenstein P, Melke J (2014). Association between ASMT and autistic-like traits in children from a Swedish nationwide cohort. Psychiatr Genet.

[26] Jha P, Sheth D, Ghaziuddin M (2007). Autism spectrum disorder and Klinefelter syndrome. Eur Child Adolesc Psychiatry.

[27] Lowe JK, Werling DM, Constantino JN, Cantor RM, Geschwind DH (2015). Social responsiveness, an autism endophenotype: genomewide significant linkage to two regions on chromosome 8. Am J Psychiatry.

[28] Hattori N, Kitada T, Matsumine H, Asakawa S, Yamamura Y, Yoshino H, Kobayashi T, Yokochi M, Wang M, Yoritaka A (1998). Molecular genetic analysis of a novel Parkin gene in Japanese families with autosomal recessive juvenile parkinsonism: evidence for variable homozygous deletions in the Parkin gene in affected individuals. Ann Neurol.

[29] Xu B, Roos JL, Levy S, van Rensburg EJ, Gogos JA, Karayiorgou M (2008). Strong association of de novo copy number mutations with sporadic schizophrenia. Nat Genet.

[30] Jarick I, Volckmar AL, Putter C, Pechlivanis S, Nguyen TT, Dauvermann MR, Beck S, Albayrak O, Scherag S, Gilsbach S (2014). Genome-wide analysis of rare copy number variations reveals PARK2 as a candidate gene for attention-deficit/hyperactivity disorder. Mol Psychiatry.

[31] Hannes FD, Sharp AJ, Mefford HC, de Ravel T, Ruivenkamp CA, Breuning MH, Fryns JP, Devriendt K, Van Buggenhout G, Vogels A (2009). Recurrent reciprocal deletions and duplications of 16p13.11: the deletion is a risk factor for MR/MCA while the duplication may be a rare benign variant. J Med Genet.

[32] Nagamani SC, Erez A, Bader P, Lalani SR, Scott DA, Scaglia F, Plon SE, Tsai CH, Reimschisel T, Roeder E (2011). Phenotypic manifestations of copy number variation in chromosome 16p13.11.. Eur J Hum Genet.

[33] Williams NM, Zaharieva I, Martin A, Langley K, Mantripragada K, Fossdal R, Stefansson H, Stefansson K, Magnusson P, Gudmundsson OO (2010). Rare chromosomal deletions and duplications in attention-deficit hyperactivity disorder: a genome-wide analysis. Lancet.

[34] Ramalingam A, Zhou XG, Fiedler SD, Brawner SJ, Joyce JM, Liu HY, Yu S (2011). 16p13.11 duplication is a risk factor for a wide spectrum of neuropsychiatric disorders. J Hum Genet.

[35] Riggs ER, Jackson L, Miller DT, Van Vooren S (2012). Phenotypic information in genomic variant databases enhances clinical care and research: the International Standards for Cytogenomic Arrays Consortium experience. Hum Mutat.

[36] Marshall CR, Noor A, Vincent JB, Lionel AC, Feuk L, Skaug J, Shago M, Moessner R, Pinto D, Ren Y (2008). Structural variation of chromosomes in autism spectrum disorder. Am J Hum Genet.

[37] Girirajan S, Dennis MY, Baker C, Malig M, Coe BP, Campbell CD, Mark K, Vu TH, Alkan C, Cheng Z (2013). Refinement and discovery of new hotspots of copy-number variation associated with autism spectrum disorder. Am J Hum Genet.

[38] Biesecker LG, Mullikin JC, Facio FM, Turner C, Cherukuri PF, Blakesley RW, Bouffard GG, Chines PS, Cruz P, Hansen NF (2009). The ClinSeq Project: piloting large-scale genome sequencing for research in genomic medicine. Genome Res.

[39] Cooper GM, Coe BP, Girirajan S, Rosenfeld JA, Vu TH, Baker C, Williams C, Stalker H, Hamid R, Hannig V (2011). A copy number variation morbidity map of developmental delay. Nat Genet.

[40] Redon R, Ishikawa S, Fitch KR, Feuk L, Perry GH, Andrews TD, Fiegler H, Shapero MH, Carson AR, Chen W (2006). Global variation in copy number in the human genome. Nature.

[41] Pinto D, Marshall C, Feuk L, Scherer SW (2007). Copy-number variation in control population cohorts. Hum Mol Genet.

[42] Perry GH, Ben-Dor A, Tsalenko A, Sampas N, Rodriguez-Revenga L, Tran CW, Scheffer A, Steinfeld I, Tsang P, Yamada NA (2008). The fine-scale and complex architecture of human copy-number variation. Am J Hum Genet.

[43] Uddin M, Thiruvahindrapuram B, Walker S, Wang Z, Hu P, Lamoureux S, Wei J, MacDonald JR, Pellecchia G, Lu C (2015). A high-resolution copy-number variation resource for clinical and population genetics. Genet Med.

[44] Suktitipat B, Naktang C, Mhuantong W, Tularak T, Artiwet P, Pasomsap E, Jongjaroenprasert W, Fuchareon S, Mahasirimongkol S, Chantratita W (2014). Copy number variation in Thai population. PLoS One.

[45] MacDonald JR, Ziman R, Yuen RK, Feuk L, Scherer SW (2014). The Database of Genomic Variants: a curated collection of structural variation in the human genome. Nucleic Acids Res.

[46] Itsara A, Cooper GM, Baker C, Girirajan S, Li J, Absher D, Krauss RM, Myers RM, Ridker PM, Chasman DI (2009). Population analysis of large copy number variants and hotspots of human genetic disease. Am J Hum Genet.

[47] Nang EEK, Khoo CM, Tai ES, Lim SC, Tavintharan S, Wong TY, Heng D, Lee J (2009). Is there a clear threshold for fasting plasma glucose that differentiates between those with and without neuropathy and chronic kidney disease? The Singapore Prospective Study Program. Am J Epidemiol.

[48] Chen P, Ong RT, Tay WT, Sim X, Ali M, Xu H, Suo C, Liu J, Chia KS, Vithana E (2013). A study assessing the association of glycated hemoglobin A1C (HbA1C) associated variants with HbA1C, chronic kidney disease and diabetic retinopathy in populations of Asian ancestry. PLoS One.

[49] Conceicao IC, Rama MM, Oliveira B, Cafe C, Almeida J, Mouga S, Duque F, Oliveira G, Vicente AM (2017). Definition of a putative pathological region in PARK2 associated with autism spectrum disorder through in silico analysis of its functional structure. Psychiatr Genet.

[50] Stefansson H, Meyer-Lindenberg A, Steinberg S, Magnusdottir B, Morgen K, Arnarsdottir S, Bjornsdottir G, Walters GB, Jonsdottir GA, Doyle OM (2014). CNVs conferring risk of autism or schizophrenia affect cognition in controls. Nature.

