# Supplementary material for: Use of clinical chromosomal microarray in Chinese patients with autism spectrum disorder—implications of a copy number variation involving DPP10
Source: Mol Autism. 2017 Jun 26;8:31. doi: 10.1186/s13229-017-0136-x (PMC5485587; doi:10.1186/s13229-017-0136-x)
Supplement: Supplementary file 2 — Coordinates [hg19] of CNVs overlapping DPP10 in chromosome 2 in Marshall et al., Girirajan et al., and the DECIPHER database. (DOCX 19 kb) [file 13229_2017_136_MOESM2_ESM.docx]

**USE OF CLINICAL CHROMOSOMAL MICROARRAY IN CHINESE PATIENTS WITH AUTISM SPECTRUM DISORDER – IMPLICATIONS OF A COPY NUMBER VARIATION INVOLVING DPP10 – Additional files**

Mak, Shui Lam1, Chiu, Ting Gee Annie^2^, Leung, Gordon Ka Chun^2^, Mak, Christopher Chun Yu^2^, Chu, Yoyo Wing Yiu^2^, Mok, Gary Tsz Kin^2^, Tang, Wing Fai^3^, Chan, Kelvin Yuen Kwong^4^, Tang, Mary Hoi Yin^3^, Lau Yim, Elizabeth Tak-Kwong^3^, So, Kin Wai^2^, Tao, Victoria Qinchen^2^, Fung, Cheuk Wing^2,5^, Wong, Virginia Chun Nei^2,5^, Uddin, Mohammed^6^, Lee, So Lun^2,5^, Marshall, Christian R.^6^, Scherer, Stephen W.^6,7^, ^#^Kan, Sik Yau Anita^4^, ^#*^Chung, Hon Yin Brian^2,3,5^

1. Department of Obstetrics and Gynaecology, Queen Elizabeth Hospital. 30 Gascoigne Road, Kowloon, Hong Kong Special Administrative Region

2. Department of Paediatrics and Adolescent Medicine, Li Ka Shing Faculty of Medicine, The University of Hong Kong. New Clinical Building, Queen Mary Hospital, 102 Pokfulam Road, Hong Kong Special Administrative Region

3. Department of Obstetrics and Gynaecology, The University of Hong Kong

4. Department of Obstetrics and Gynaecology, Tsan Yuk Hospital. 30 Hospital Road, Sai Ying Pun, Hong Kong Special Administrative Region

5. Duchess of Kent Children's Hospital. 12 Sandy Bay Road, Pokfulam, Hong Kong Special Administrative Region

6. The Centre for Applied Genomics, The Hospital for Sick Children, 555 University Ave, Toronto, Canada

7. McLaughlin Centre and Department of Molecular Genetics, University of Toronto, Toronto, Canada

#Co-corresponding author

*Corresponding author: Dr Hon-Yin Brian Chung, [bhychung@hku.hk](mailto:bhychung@hku.hk), +852 2255 4482

**Supplementary Table S2. Coordinates [hg19] of CNVs overlapping *DPP10* in chromosome 2 in Marshall *et al.,* Girirajan *et al.***

| Accession numbers | Start coordinates | End coordinates | CNVs size | Duplication or deletion | No. with the CNVs |
| --- | --- | --- | --- | --- | --- |
| *Marshall et al.* | | | | | |
| SK0147-003 | 115139326 | 115617696 | 478370 | Deletion | 1 |
| SK0288-003 | 115425410 | 115530530 | 105120 | Duplication | 1 |
| *Girirajan et al.* | | | | | |
| 11033.pl | 116094410 | 116985952 | 891542 | Duplication | 1 |
| 11324.p1 | 116544645 | 116573813 | 29168 | Duplication | 1 |
| 13103.p1 | 116525224 | 116677847 | 152623 | Duplication | 1 |
| 13418.p1 | 116525224 | 116677847 | 152623 | Duplication | 1 |
| 14013.p1 | 116525224 | 116677847 | 152623 | Duplication | 1 |
